# Alcohol Policies and Alcoholic Cirrhosis Mortality in the United States

**DOI:** 10.5888/pcd12.150200

**Published:** 2015-10-15

**Authors:** Scott E. Hadland, Ziming Xuan, Jason G. Blanchette, Timothy C. Heeren, Monica H. Swahn, Timothy S. Naimi

**Affiliations:** Author Affiliations: Ziming Xuan, Jason G. Blanchette, Timothy C. Heeren, Boston University School of Public Health, Department of Community Health Sciences, Boston, Massachusetts; Monica H. Swahn, School of Public Health, Georgia State University, Atlanta, Georgia; Timothy S. Naimi, Boston University School of Public Health, Department of Community Health Sciences, and Boston Medical Center, Section of General Internal Medicine, Boston, Massachusetts. Dr Hadland is also affiliated with Harvard Medical School, Department of Pediatrics, Boston, Massachusetts.

## Abstract

**Introduction:**

Stronger alcohol policies predict decreased alcohol consumption and binge drinking in the United States. We examined the relationship between the strength of states’ alcohol policies and alcoholic cirrhosis mortality rates.

**Methods:**

We used the Alcohol Policy Scale (APS), a validated assessment of policies of the 50 US states and Washington DC, to quantify the efficacy and implementation of 29 policies. State APS scores (theoretical range, 0–100) for each year from 1999 through 2008 were compared with age-adjusted alcoholic cirrhosis death rates that occurred 3 years later. We used Poisson regression accounting for state-level clustering and adjusting for race/ethnicity, college education, insurance status, household income, religiosity, policing rates, and urbanization.

**Results:**

Age-adjusted alcoholic cirrhosis mortality rates varied significantly across states; they were highest among males, among residents in states in the West census region, and in states with a high proportion of American Indians/Alaska Natives (AI/ANs). Higher APS scores were associated with lower mortality rates among females (adjusted incidence rate ratio [IRR], 0.91 per 10-point increase in APS score; 95% confidence interval [95% CI], 0.84–0.99) but not among males (adjusted IRR, 0.97; 95% CI, 0.90–1.04). Among non-AI/AN decedents, higher APS scores were also associated with lower alcoholic cirrhosis mortality rates among both sexes combined (adjusted IRR, 0.89; 95% CI, 0.82–0.97). Policies were more strongly associated with lower mortality rates among those living in the Northeast and West census regions than in other regions.

**Conclusions:**

Stronger alcohol policy environments are associated with lower alcoholic cirrhosis mortality rates. Future studies should identify underlying reasons for racial/ethnic and regional differences in this relationship.

## Introduction

Excessive alcohol consumption is the third leading preventable cause of death in the United States; alcoholic cirrhosis accounts for nearly half of all cirrhosis deaths (1,2). Although the age-adjusted alcoholic cirrhosis mortality rate in the United States is lower than the global average (3.3 compared with 5.9 deaths per 100,000 per year), it has steadily and significantly increased during the past decade (1). Moreover, significant differences exist among demographic subgroups; death rates are particularly high among American Indians/Alaska Natives (AI/ANs) (3).

Stronger state-level alcohol policies have been linked to decreased binge drinking (4,5). State alcohol policies include diverse interventions such as implementing alcohol excise taxes; regulating the location, time, and quantity of alcohol sales; and restricting retail signage and media advertising (6,7). To date, most studies of alcohol-related outcomes have examined single policy changes (8–11). However, alcohol consumption is simultaneously affected by multiple policies of varying efficacy and levels of implementation. Therefore, it is critical to consider states’ overall alcohol policy environments. Furthermore, analyses should carefully examine subpopulations, including AI/ANs, who have high alcohol-related mortality rates and may live in autonomous regions where policies may have poor penetration (3,12).

Understanding alcohol policy environments and their links to health may inform policy makers who seek to reduce alcohol-related harm. To examine the association between policies and alcoholic cirrhosis mortality rates, we used the Alcohol Policy Scale (APS) score, which quantifies states’ alcohol policy environments by considering not only the efficacy of policies but also their degree of implementation (4). We hypothesized that stronger policy environments would be associated with lower alcoholic cirrhosis mortality rates but that this association might not be as robust for AI/ANs.

## Methods

The Alcohol Policy Scale (APS) is a validated assessment of alcohol policies of the 50 US states and Washington, DC, that was described previously (4,6). Briefly, the APS integrated ratings on the efficacy and degree of implementation of 29 policies collected and assessed by a Delphi panel. Panel members were experts from academia, government, and the private sector and represented a range of disciplines, including law, epidemiology, psychology, sociology, and economics.

Each panelist independently nominated alcohol policies considered to effectively reduce excessive drinking or alcohol-related harm. The primary source for policies was the Alcohol Policy Information System maintained by the National Institute on Alcohol Abuse and Alcoholism (13). Examples of policies incorporated into a state’s APS score included alcohol taxes, retail price restrictions, and hours of sales regulations; a full list is available elsewhere (6). The APS incorporated information on the efficacy and legislative implementation of each alcohol policy.

To determine the efficacy of each policy, the study investigators generated idealized descriptions for each policy. For each state-year, panelists independently rated the efficacy of each policy for reducing binge drinking in the general population on the basis of their expertise and available scientific evidence. Ratings were on a 5-point Likert scale (1 = low efficacy to 5 = high efficacy) and provided for each state and year. (A separate ratings process examined policy efficacy for reducing binge drinking among youths and alcohol-impaired driving, but these efficacy ratings were not considered in these analyses.) Next, panelists met to review the combined ratings and discuss their individual ratings, focusing on the strength of the scientific literature supporting each policy. Afterward, each panelist again independently rated each policy a second time concerning reducing binge drinking in the general population. These final ratings comprised the efficacy component of the APS.

To determine the implementation of each alcohol policy, study investigators generated an implementation rating scale on the basis of the statutory design of each policy, specifically considering provisions that made the policy broadly applicable, effective, or enforceable (4). Panelists then provided feedback on the implementation rating scales, which were subsequently revised by the study investigators. Then the scales were applied to all policies for each state and year, with a range of 0 (no policy) to 1.0 (policy with full implementation).

Ultimately, 29 policies were included in the APS. To generate an overall APS score for each state and year, each efficacy score was multiplied by its implementation score and summed with all other policies for that state and year (4,6). Scores were standardized such that the theoretical range of the APS score was from 0 to 100; high APS scores represented strong alcohol policy environments. APS scores from 1999 through 2008 were used in these analyses.

Age-adjusted alcoholic cirrhosis mortality rates were obtained from the Centers for Disease Control and Prevention (CDC) Wide-Ranging Online Data for Epidemiologic Research (WONDER) system. Using the *International Classification of Diseases, 10th Revision, Clinical Modification* code K70.x (alcoholic liver disease), we extracted age-adjusted mortality data for all 50 states and Washington, DC, for all years from 2002 through 2011, and for males and females separately, resulting in 1,020 observations (14). The range 2002 through 2011 was selected to allow for a 3-year lag between APS score and associated mortality rates. Of the 1,020 state-year observations, 123 (12.1%) were suppressed by the CDC WONDER system because of insufficient counts for that state-year to preserve confidentiality. In such cases, a 2-year running average was used, such that the overall mortality rate in a state for a suppressed year was combined with the rate from the previous year, which usually provided sufficient counts so that the rate was no longer suppressed. By this process, 105 (10.3%) missing mortality rates were imputed, leaving only 18 (1.8%) state-year observations suppressed.

We examined age-adjusted rates for 2010–2011, the most recent years analyzed, and generated a map comparing APS scores and age-adjusted alcoholic cirrhosis mortality rates for both sexes combined to examine geographical differences. Because regional differences in mortality rates were apparent after constructing this map, we examined age-adjusted mortality rates by sex according to US census region (region 1 [Northeast], region 2 [Midwest], region 3 [South], region 4 [West]). Because we hypothesized that the association between APS and mortality rate might be modified by race/ethnicity, we also examined mortality rates by sex according to states’ proportion of racial/ethnic subgroups (non-Hispanic white, non-Hispanic black, Hispanic, and AI/AN [including Hispanic AI/AN]).

We then examined the association between APS and mortality rates. Each state-year of mortality data was matched to the corresponding APS using a 3-year lag. For example, states’ APS scores from 1999 were related to mortality rates for 2002. The 3-year lag was used to increase the number of overlapping years of APS scores and mortality data (compared with a longer lag, which would have resulted in fewer overlapping years of data). This shorter lag period was consistent with evidence showing that alcohol policy changes may demonstrate an early effect on cirrhosis death rates (15–17).

Analyses used Poisson regression with robust standard error estimates to account for state-level clustering (18). Incidence rate ratios (IRRs) were adjusted for state-level race/ethnicity, college education, insurance status, household income, religiosity, policing rates, level of urbanization, and study year as an indicator variable. Covariates were extracted from the US Census Bureau’s American Community Survey and Current Population Survey and were time-updated to match the mortality rate for each state-year observation (19–21). As a sensitivity analysis, we also repeated all models using a fixed effects approach in which we included an indicator variable for every state and repeated all models using a random intercepts approach in which state was a level 2 variable; results were consistent with the findings from the robust sandwich standard error estimates (not shown).

To examine regional differences in the association between APS and alcoholic cirrhosis mortality rates, we also conducted analyses stratified by census region. Recognizing that AI/ANs in many places in the United States have elevated alcohol-related mortality rates (3) and that state health policies may not penetrate sovereign or physically remote AI/AN locales (12), we also stratified analyses according to states with proportion of AI/ANs (≥2.5% or <2.5%). Proportions were time-updated during the study period. We also repeated analyses using mortality data extracted from CDC WONDER that excluded AI/AN decedents. Finally, we generated scatterplots of states’ proportion of AI/ANs and alcoholic cirrhosis mortality rates according to sex, using the most recent years of data examined (2010–2011).

Analyses were performed using STATA version 13/SE (StataCorp LP). All *P* values were 2-sided, and tests were considered significant at *P* < .05.

## Results

For all states from 1999 through 2008, the mean APS score was 41 (range, 23–66) and increased during the study period (mean APS in 1999, 38; mean APS in 2008, 42; *P* < .001). The mean age-adjusted alcoholic cirrhosis mortality rate for the United States from 2002 through 2011 was 4.7 deaths per 100,000 per year (95% confidence interval [CI], 4.2–5.3) with a range of 2.5 (Pennsylvania) to 10.3 (New Mexico). Alcoholic cirrhosis mortality rates increased during the study period (mean age-adjusted mortality rate in 2002 was 4.6 deaths per 100,000; mean rate in 2011 was 5.3 deaths per 100,000; *P* < .001).

**Figure 1:**
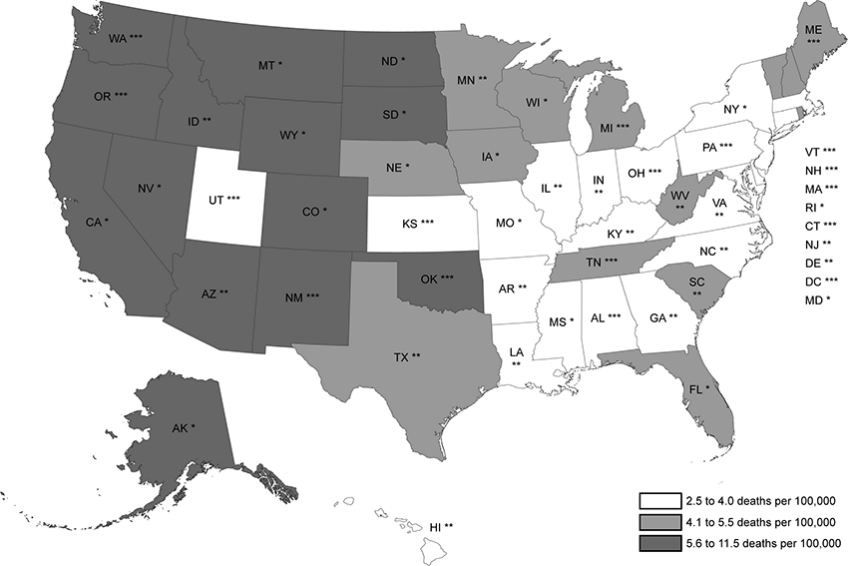
State age-adjusted alcoholic cirrhosis mortality rates (men and women combined) and associated alcohol policy score tertiles. Mortality rates from 2010–2011 were compared with alcohol policy scores from 2008 to introduce a 2- to 3-year lag. States with the highest tertile of alcohol policy score (ie, strongest policy environment) are marked by 3 asterisks (***); the middle tertile, by 2 asterisks (**); and the lowest tertile, by a single asterisk (*).

During the 2 most recent years studied (2010–2011), age-adjusted alcoholic cirrhosis mortality rates for males were significantly higher than those for females, and there was substantial variability between states (Table 1). Figure 1 shows states’ mortality rates and APS score tertiles for both sexes combined. In general, there was considerable geographic variation in alcoholic cirrhosis mortality rates; rates among states in the West census region were higher than among those in other US census regions.

**Table 1:** Age-Adjusted Alcoholic Cirrhosis Mortality Rates^a^ Per 100,000 Population Per Year, by State, United States, 2010–2011

State	Overall	Males	Females
	Rate (95% Confidence Interval)		
US average	4.8 (4.7–4.8)	7.0 (6.9–7.1)	2.7 (2.6–2.8)
New Mexico	11.5 (10.5–12.6)	16.4 (14.6–18.1)	7.1 (5.9–8.2)
Arizona	9.3 (8.7–9.8)	13.1 (12.3–14.0)	5.8 (5.2–6.3)
Wyoming	8.7 (7.0–10.5)	10.8 (8.2–13.8)	6.7 (4.7–9.3)
Oregon	8.5 (7.9–9.2)	11.7 (10.7–12.7)	5.6 (4.9–6.3)
California	8.2 (8.0–8.5)	12.3 (11.9–12.6)	4.6 (4.4–4.8)
Washington	8.2 (7.7–8.7)	11.0 (10.2–11.8)	5.6 (5.0–6.1)
South Dakota	8.1 (6.7–9.5)	10.3 (8.2–12.8)	6.1 (4.5–8.1)
North Dakota	7.8 (6.3–9.3)	10.4 (8.1–13.1)	5.2 (3.6–7.4)
Montana	7.7 (6.5–8.9)	10.3 (8.4–12.2)	5.2 (3.9–6.8)
Colorado	7.5 (7.0–8.1)	10.5 (9.6–11.4)	4.8 (4.2–5.4)
Oklahoma	7.5 (6.9–8.1)	10.3 (9.3–11.3)	4.9 (4.2–5.6)
Alaska	7.4 (6.0–8.9)	8.0 (6.1–10.3)	6.8 (5.0–8.9)
Idaho	7.2 (6.3–8.1)	8.8 (7.3–10.3)	5.7 (4.6–7.0)
Nevada	6.6 (5.9–7.2)	8.3 (7.2–9.3)	4.8 (4.0–5.7)
Vermont	5.5 (4.4–6.8)	7.7 (5.8–10.0)	3.4 (2.2–5.0)
Florida	5.1 (4.9–5.3)	7.5 (7.1–7.9)	3.0 (2.8–3.2)
Nebraska	5.1 (4.4–5.8)	6.7 (5.6–7.9)	3.7 (2.8–4.6)
Rhode Island	4.9 (4.0–5.7)	7.6 (6.1–9.3)	2.5 (1.7–3.5)
Michigan	4.8 (4.5–5.1)	6.9 (6.4–7.4)	2.9 (2.6–3.2)
District of Columbia	4.7 (3.5–6.1)	6.1 (4.2–8.5)	3.4 (2.1–5.2)
South Carolina	4.6 (4.2–5.0)	7.3 (6.5–8.0)	2.3 (1.9–2.7)
Tennessee	4.6 (4.3–5.0)	7.0 (6.4–7.7)	2.4 (2.0–2.7)
Maine	4.5 (3.8–5.2)	6.3 (5.1–7.6)	2.9 (2.2–3.8)
Wisconsin	4.5 (4.1–4.9)	6.3 (5.6–6.9)	2.8 (2.3–3.2)
Iowa	4.4 (3.9–4.9)	6.4 (5.5–7.3)	2.5 (2.0–3.1)
Texas	4.3 (4.2–4.5)	6.7 (6.4–7.1)	2.1 (1.9–2.3)
New Hampshire	4.2 (3.5–4.9)	6.3 (5.1–7.6)	2.4 (1.7–3.3)
West Virginia	4.2 (3.5–4.8)	6.5 (5.4–7.6)	1.9 (1.4–2.6)
Minnesota	4.1 (3.7–4.5)	5.5 (4.9–6.1)	2.8 (2.3–3.2)
Ohio	4.1 (3.8–4.3)	6.1 (5.7–6.6)	2.2 (1.9–2.4)
Kansas	4.0 (3.5–4.5)	6.1 (5.2–7.0)	2.0 (1.6–2.6)
Hawaii	4.0 (3.3–4.7)	6.0 (4.8–7.4)	2.1 (1.4–2.9)
North Carolina	4.0 (3.7–4.2)	6.2 (5.7–6.7)	2.0 (1.7–2.3)
Indiana	3.9 (3.6–4.2)	6.0 (5.4–6.5)	2.0 (1.7–2.3)
New York	3.4 (3.2–3.6)	5.3 (4.9–5.6)	1.7 (1.6–1.9)
Utah	3.4 (2.8–3.9)	4.5 (3.6–5.4)	2.3 (1.8–3.0)
Kentucky	3.2 (2.8–3.7)	4.8 (4.0–5.6)	1.7 (1.3–2.3)
Arkansas	3.2 (2.9–3.6)	5.3 (4.6–6.0)	1.4 (1.0–1.7)
Missouri	3.2 (2.9–3.5)	4.7 (4.2–5.3)	1.7 (1.4–2.0)
Illinois	3.1 (2.9–3.4)	4.5 (4.2–4.9)	1.9 (1.6–2.1)
Mississippi	3.0 (2.6–3.4)	5.0 (4.2–5.8)	1.2 (0.9–1.7)
New Jersey	2.9 (2.7–3.2)	4.4 (3.9–4.8)	1.7 (1.4–1.9)
Georgia	2.9 (2.7–3.2)	4.6 (4.2–5.0)	1.4 (1.2–1.6)
Massachusetts	2.9 (2.6–3.2)	4.4 (3.9–4.9)	1.5 (1.2–1.8)
Alabama	2.9 (2.5–3.2)	4.6 (4.0–5.2)	1.4 (1.1–1.7)
Delaware	2.8 (2.1–3.6)	4.7 (3.4–6.2)	NA^b^
Pennsylvania	2.8 (2.6–3.0)	4.0 (3.7–4.4)	1.6 (1.4–1.8)
Virginia	2.7 (2.4–2.9)	3.9 (3.5–4.3)	1.6 (1.4–1.9)
Connecticut	2.7 (2.3–3.0)	3.9 (3.3–4.6)	1.6 (1.2–2.0)
Louisiana	2.7 (2.3–3.0)	4.0 (3.4–4.6)	1.5 (1.1–1.8)
Maryland	2.5 (2.2–2.8)	3.7 (3.2–4.2)	1.4 (1.2–1.7)

Abbreviations: CDC, Centers for Disease Control and Prevention; NA, not applicable; WONDER, Wide-Ranging Online Data for Epidemiologic Research.

a All age-adjusted rates obtained from CDC WONDER database and are averaged for 2010–2011.

b Mortality data for females in Delaware were not available due to insufficient counts in CDC WONDER database.

Mean age-adjusted alcoholic cirrhosis mortality rates during the entire study period (2002–2011) were analyzed according to racial/ethnic group and census region (Table 2). In every comparison, mortality rates were significantly higher among males than among females. Significant differences in mortality rates were noted between females from states with differing proportion of non-Hispanic blacks (≥5% and <5%) and between states with differing proportions of AI/ANs (≥2.5% and <2.5%) for both sexes. Region 4 (West) had significantly higher mortality rates than other regions for both sexes.

**Table 2:** Mean Age-Adjusted Alcoholic Cirrhosis Mortality Rates Per 100,000 Population Per Year, by Mean State-Level Characteristic, United States, 2002–2011

State Characteristic	Mortality Per 100,000 Per Year (95% CI)		
	Overall	Males	Females
**All races/ethnicities and regions**	4.5 (3.5–5.5)	6.7 (5.2–8.2)	2.4 (1.9–2.9)
**% Non-Hispanic white**			
<80	4.8 (3.4–6.3)	7.2 (5.1–9.4)	2.5 (1.7–3.3)
≥80	4.0 (3.4–4.6)	5.8 (5.1–6.6)	2.2 (1.8–2.6)
**% Non-Hispanic black**			
<5	5.9 (4.8–6.9)	8.3 (6.7–9.9)	3.5 (2.8–4.1)
≥5	4.1 (3.1–5.1)	6.1 (4.6–7.7)	2.0 (1.5–2.6)
**% Hispanic**			
<5	3.9 (3.3–4.4)	5.8 (5.0–6.7)	2.0 (1.7–2.3)
≥5	5.6 (4.1–7.0)	8.1 (6.0–10.3)	3.0 (2.3–3.8)
**% American Indian/Alaska Native**			
<2.5	4.2 (3.1–5.3)	6.3 (4.7–8.0)	2.2 (1.6–2.8)
≥2.5	7.4 (6.7–8.0)	10.2 (9.3–11.1)	4.5 (4.1–5.0)
**Census region**			
1 (Northeast)	3.0 (2.7–3.3)	4.5 (4.0–5.0)	1.5 (1.4–1.7)
2 (Midwest)	3.7 (3.3–4.1)	5.4 (4.8–6.0)	2.0 (1.8–2.3)
3 (South)	3.8 (3.3–4.4)	5.9 (5.1–6.6)	1.9 (1.5–2.2)
4 (West)	7.6 (6.9–8.3)	10.9 (9.5–12.3)	4.2 (4.0–4.5)

Abbreviation: CI, confidence interval.

In unadjusted analyses, APS score was not significantly associated with alcoholic cirrhosis mortality when both sexes were considered together (IRR, 0.93 per 10-point increase in APS score; 95% CI, 0.80–1.07) and separately (male IRR, 0.95; 95% CI, 0.83–1.08; female IRR, 0.86; 95% CI, 0.72–1.04). After adjusting for covariates, higher APS score was significantly associated with lower alcoholic cirrhosis mortality rates among females but not among males, and among the overall population when both sexes were combined (Table 3).

**Table 3:** Incidence Rate Ratios (IRRs) for Association of Alcohol Policy Scale (APS) Score^a^ With State-Level, Age-Adjusted Alcoholic Cirrhosis Mortality Rates, by US Census Region and by State Proportion of American Indians/Alaska Natives (AI/ANs), 2002–2011

Subcategory	Adjusted IRR (95% Confidence Interval)^b^		
	Overall	Males	Females
**All races/ethnicities^c^**	0.95 (0.88–1.03)	0.97 (0.90–1.04)	0.91 (0.84–0.99)
**Census region^d^**			
1 (Northeast)	0.68 (0.60–0.78)	0.70 (0.60–0.82)	0.61 (0.58–0.65)
2 (Midwest)	1.01 (0.93–1.09)	1.02 (0.96–1.09)	0.96 (0.81–1.14)
3 (South)	0.87 (0.76–0.99)	0.87 (0.77–0.98)	0.82 (0.71–0.95)
4 (West)	0.87 (0.73–1.03)	0.89 (0.75–1.05)	0.83 (0.68–1.00)
**AI/AN^d^^,^ e, %**			
<2.5	0.90 (0.82–0.98)	0.90 (0.82–0.98)	0.88 (0.81–0.96)
≥2.5	0.98 (0.89–1.08)	0.99 (0.90–1.08)	0.98 (0.86–1.11)
**All races/ethnicities, excluding AI/AN decedents^c^**	0.89 (0.82–0.97)	0.94 (0.87–1.01)	0.82 (0.69–0.98)
**Census region**			
1 (Northeast)	0.55 (0.43–0.72)	0.71 (0.60–0.84)	0.39 (0.25–0.61)
2 (Midwest)	0.99 (0.92–1.07)	1.01 (0.95–1.08)	0.95 (0.80–1.12)
3 (South)	0.79 (0.59–1.06)	0.91 (0.78–1.06)	0.59 (0.35–1.01)
4 (West)	0.89 (0.80–0.99)	0.83 (0.70–0.99)	0.96 (0.85–1.09)

a A 3-year lag was introduced between APS score and mortality rates (eg, states’ mortality rates from 2002 were associated with APS scores from 1999).

b IRR reported per 10-point increase in APS score; models adjusted for year, race/ethnicity, college education, insurance status, household income, religiosity, policing rates, and proportion living in rural/urban areas.

c IRR is adjusted for all covariates as well as census region and proportion of AI/ANs.

d Model stratified by census region is adjusted for all covariates, as well as proportion of AI/ANs; model stratified by proportion of AI/ANs is adjusted for all covariates, as well as census region.

e Includes 806 state-year observations with the proportion of AI/ANs <2.5% and 214 observations with proportion ≥2.5%.

Additionally, significant geographical variation was seen in the association between APS score and alcoholic cirrhosis mortality rates. For both sexes, APS score was significantly associated with decreased mortality rates in the Northeast and South census regions, but not in the Midwest or West regions. Furthermore, the association of APS score with lower mortality rates was present in states with less than 2.5% of AI/ANs but not in states with 2.5% or more AI/ANs. When analyses were repeated excluding all AI/AN decedents, geographical variation was again present in the association between APS score and alcoholic cirrhosis mortality rates. For both sexes combined, APS score was significantly associated with decreased mortality rates in the Northeast and West, a finding that also held when males were analyzed separately; when females were analyzed separately, the association was significant only in the Northeast.

**Figure 2:**
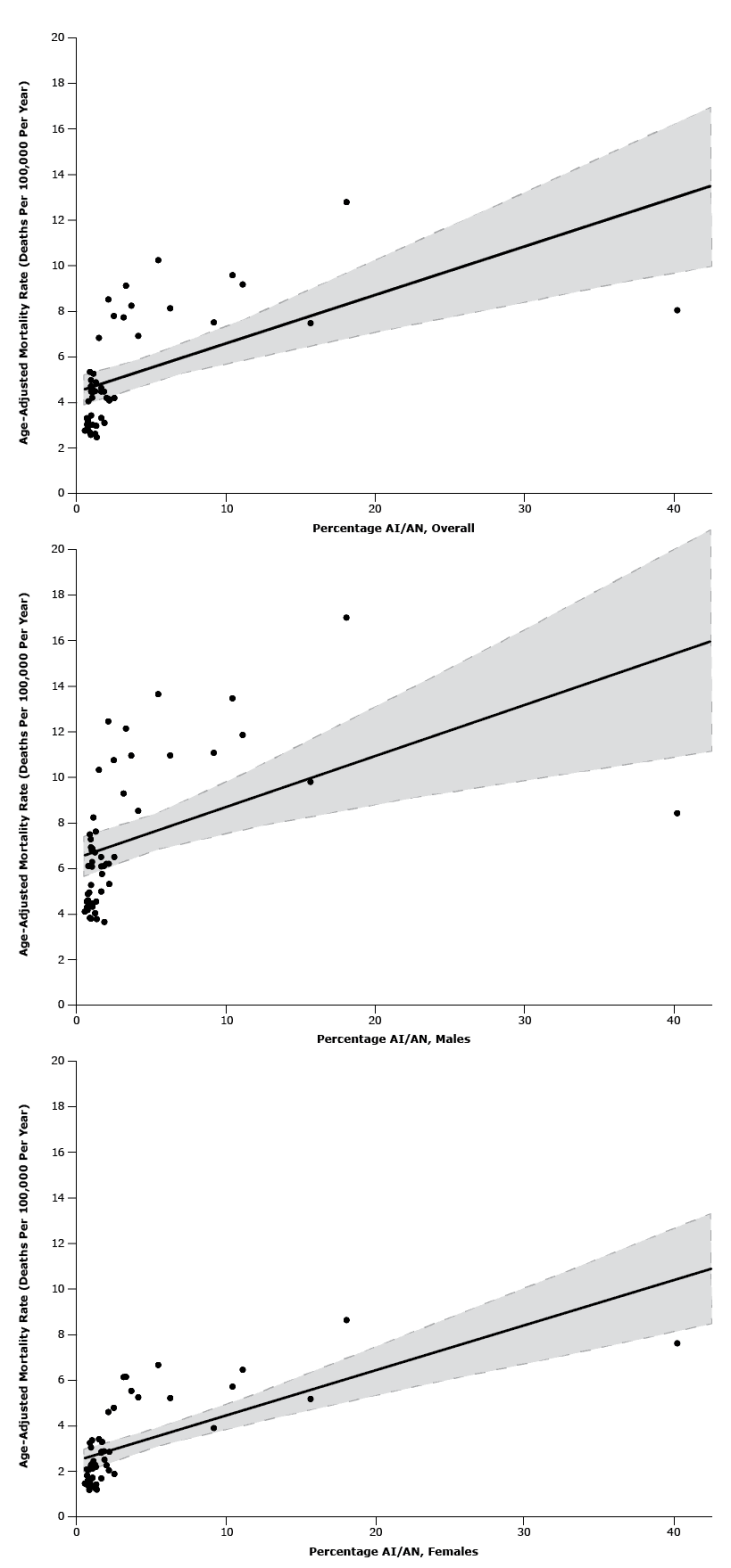
Relationship between proportion of American Indians/Alaska Natives in states’ populations and age-adjusted alcoholic cirrhosis mortality rates of states among (2a) both sexes [*r* ^2^ = 0.320, *P* < .001], (2b) males [*r* ^2^ = 0.473, *P* < .001], and (2c) females [*r* ^2^ = 0.217, *P* = .001], United States, 2010–2011. Solid line represents curve of best fit determined by the least-squares method. Hashed lines represent upper and lower 95% confidence intervals.

Scatterplots of state age-adjusted alcoholic cirrhosis mortality rates by the proportion of AI/ANs are depicted in Figure 2. In general, as the proportion of AI/ANs increased, mortality rates increased.

## Discussion

We found that stronger state alcohol policy environments were associated with lower alcoholic cirrhosis mortality rates for certain regions and racial/ethnic groups in the United States. Using the APS score, which quantifies a state’s alcohol policy environment, we found that a 10-point increase (ie, a stronger policy environment) was associated with a 9% decrease in alcoholic cirrhosis mortality among females. To provide context to this 10-point difference, the range of APS scores observed across states during the study period was 23 to 66, so there is notable between-state variation in the strength of alcohol policy environments. Examining mortality rates among non-AI/AN decedents indicated that a 10-point increase in APS score was associated with an 11% decrease in mortality among both sexes, a finding driven primarily by females, whose mortality rate decreased by 18% for a 10-point increase in APS score.

These findings are consistent with the recent finding that stronger state alcohol policy environments in the United States are associated with decreased binge drinking prevalence (4). Coupled with data suggesting that alcohol consumption is tied to alcoholic cirrhosis mortality at the aggregate level (7,15,22), our results are consistent with a potentially protective role for alcohol policies in reducing cirrhosis mortality. State alcohol policies aimed at reducing alcohol consumption in the general population are more strongly associated with reduced binge drinking than are more targeted policies, such as policies focusing solely on underage drinking or impaired driving (5). Tax and price policies appear to be particularly effective for reducing adult binge drinking as well as alcoholic cirrhosis mortality rates and are likely critical components of a robust alcohol policy environment (5,7,11,23,24).

Although alcoholic cirrhosis mortality has a long lag period, lasting up to decades of alcohol exposure (25), our findings showed a significant association between alcohol policy environments and mortality rates using only a 3-year lag. At the aggregate level, however, there may be an immediate effect on cirrhosis mortality demonstrated after sudden declines in alcohol consumption (7,15,17,25). As suggested previously (7,17), at any given time, there exists a reservoir of individuals with a prior and extensive history of drinking and alcoholic cirrhosis who are approaching a mortality threshold. If per capita alcohol consumption increases, a large number of those individuals are at greater risk of death in the near future; if per capita alcohol consumption decreases, those same individuals are at lower risk of immediate mortality. This phenomenon was observed historically after the institution and subsequent repeal of prohibition in the United States (15). Our results support that strengthening alcohol policy environments may be beneficial for the overall population in the short-term, despite the long lag between alcohol consumption and death from cirrhosis observed at the individual level.

The association of strong alcohol policy environments with mortality rate in the overall population was driven primarily by the death rate among females. Alcohol policy environments may have a greater effect on excess alcohol consumption among females than among males (5,26). We also noted that alcohol policy environments were not as strongly associated with reduced alcoholic cirrhosis mortality rates when AI/AN decedents were included in analyses. Our findings are consistent with evidence demonstrating elevated rates of alcohol-related death among AI/AN populations relative to other US populations (3) and suggest that changes in alcohol policies may be less protective for AI/AN individuals who live in autonomous or physically remote regions in which state alcohol policies have poor penetration (12). Additionally, our study did not examine the potential protective role of tribal alcohol policies, an area that requires further study (27,28).

Finally, we noted significant regional variation in the relationship between alcohol policy environment and mortality rates, with the strongest association noted in the Northeast census region. Other studies have shown significant regional variation in the effects of alcohol policy on drinking-related behaviors (4,9,10,15,26,29), including a recent assessment of alcohol poisoning deaths in the United States that showed high mortality in the West census region relative to the Northeast and South (30). It is possible that enforcement of alcohol policies may vary across regions and contribute to the differences in effect sizes observed in our study or that proximity to care varies among states. Future studies should continue to assess the differential effects of alcohol policies on other health concerns that are linked to excessive alcohol use for specific population subgroups according to sex, race/ethnicity, and geographical location.

This study has several limitations. Although we carefully examined the efficacy and implementation of alcohol policies, the APS score does not incorporate enforcement of policies given the absence of any reliable publicly available data on enforcement across states. Nonetheless, when designing the APS score, study investigators and the expert panel incorporated into the implementation ratings policy provisions that made policies more likely to be enforceable (4,6). Our analyses also controlled for number of police officers per capita as a state-level covariate to help account for variations in enforcement.

Additionally, despite using a lag period between exposure and outcome and controlling for year, there remains the possibility of reverse causation between APS score and alcoholic cirrhosis mortality. Specifically, states that had lower consumption of alcohol may have been the same states that implemented strong alcohol policy environments. We introduced a 3-year lag between a state’s APS score and associated mortality rate to allow time for state policies to be implemented and enforced; this lag helps to establish temporality, even if causation cannot be fully established. We also cannot exclude residual confounding despite controlling for a range of state-level covariates. We exclusively studied alcoholic cirrhosis and did not examine the contribution of other potentially overlapping causes of cirrhosis, such as hepatitis C. Finally, although the APS incorporates state-level alcohol policies, it does not include federal or local policies (eg, federal regulations on alcohol marketing, county or municipal taxes on alcohol purchases, tribal alcohol policies).

This study established an association between the strength of states’ alcohol policy environments and mortality from a leading alcohol-attributable cause of death. Future studies should further assess the strength and directionality of this association. Our findings suggest that strengthening alcohol policy environments may be an effective intervention for reducing cirrhosis mortality. Researchers and policy makers should additionally consider whether alcohol policies are uniformly protective for all citizens, given that significant disparities in mortality rates persist for AI/ANs relative to others.

## References

[1] National Center for Chronic Disease Prevention and Health Promotion. Excessive alcohol use: addressing a leading risk for death, chronic disease, and injury. http://www.cdc.gov/chronicdisease/resources/publications/aag/pdf/2011/alcohol_aag_web_508.pdf. Accessed February 16, 2015.

[2] Rehm J , Samokhvalov AV , Shield KD . Global burden of alcoholic liver diseases. J Hepatol 2013;59(1):160–8. 10.1016/j.jhep.2013.03.007 23511777

[3] Centers for Disease Control and Prevention (CDC). Alcohol-attributable deaths and years of potential life lost among American Indians and Alaska Natives — United States, 2001–2005. MMWR Morb Mortal Wkly Rep 2008;57(34):938–41. 18756193

[4] Naimi TS , Blanchette J , Nelson TF , Nguyen T , Oussayef N , Heeren TC , A new scale of the U.S. alcohol policy environment and its relationship to binge drinking. Am J Prev Med 2014;46(1):10–6. 10.1016/j.amepre.2013.07.015 24355666 PMC3878154

[5] Xuan Z , Blanchette J , Nelson TF , Heeren T , Oussayef N , Naimi TS . The alcohol policy environment and policy subgroups as predictors of binge drinking measures among US adults. Am J Public Health 2015;105(4):816–22. 10.2105/AJPH.2014.302112 25122017 PMC4358175

[6] Nelson TF , Xuan Z , Babor TF , Brewer RD , Chaloupka FJ , Gruenewald PJ , Efficacy and the strength of evidence of U.S. alcohol control policies. Am J Prev Med 2013;45(1):19–28. 10.1016/j.amepre.2013.03.008 23790985 PMC3708657

[7] Cook PJ , Tauchen G . The effect of liquor taxes on heavy drinking. Bell J Econ 1982;13(2):379–90. 10.2307/3003461

[8] Fell JC , Fisher DA , Voas RB , Blackman K , Tippetts AS . The impact of underage drinking laws on alcohol-related fatal crashes of young drivers. Alcohol Clin Exp Res 2009;33(7):1208–19. 10.1111/j.1530-0277.2009.00945.x 19389192 PMC2825167

[9] Wagenaar AC , Maldonado-Molina MM , Wagenaar BH . Effects of alcohol tax increases on alcohol-related disease mortality in Alaska: time-series analyses from 1976 to 2004. Am J Public Health 2009;99(8):1464–70. 10.2105/AJPH.2007.131326 19008507 PMC2707462

[10] Wagenaar AC , Toomey TL . Effects of minimum drinking age laws: review and analyses of the literature from 1960 to 2000. J Stud Alcohol Suppl 2002;(14):206–25. 10.15288/jsas.2002.s14.206 12022726

[11] Ponicki WR , Gruenewald PJ . The impact of alcohol taxation on liver cirrhosis mortality. J Stud Alcohol 2006;67(6):934–8. 10.15288/jsa.2006.67.934 17061012

[12] Bryan RT , Schaefer RM , DeBruyn L , Stier DD . Public health legal preparedness in Indian country. Am J Public Health 2009;99(4):607–14. 10.2105/AJPH.2008.146522 19150897 PMC2661496

[13] National Institute on Alcohol Abuse and Alcoholism. Alcohol Policy Information System. http://alcoholpolicy.niaaa.nih.gov/. Accessed February 10, 2015.

[14] Centers for Disease Control and Prevention. CDC WONDER. http://wonder.cdc.gov/. Accessed February 10, 2015.

[15] Cook P . Paying the tab: the economics of alcohol policy. Princeton (NJ): Princeton University Press; 2007.

[16] Ledermann S . Alcool, alcoolisme, alcoolisation. Inst Natl D’etudes Demogr 1956;11:531–40.

[17] Norström T , Skog OJ . Alcohol and mortality: methodological and analytical issues in aggregate analyses. Addiction 2001;96(Suppl 1):S5–17. 10.1080/09652140020021143 11228078

[18] Liang K-Y , Zeger SL . Longitudinal data analysis using generalized linear models. Biometrika 1986;73(1):13–22. 10.1093/biomet/73.1.13

[19] Xuan Z , Nelson TF , Heeren T , Blanchette J , Nelson DE , Gruenewald P , Tax policy, adult binge drinking, and youth alcohol consumption in the United States. Alcohol Clin Exp Res 2013;37(10):1713–9. 23711219 10.1111/acer.12152 PMC3795905

[20] US Census Bureau. Current Population Survey. http://www.census.gov/cps/. Accessed February 10, 2015.

[21] US Census Bureau. American Community Survey. http://www.census.gov/acs/www/data_documentation/data_main/. Accessed February 10, 2015.

[22] Ramstedt M . Alcohol consumption and liver cirrhosis mortality with and without mention of alcohol—the case of Canada. Addiction 2003;98(9):1267–76. 10.1046/j.1360-0443.2003.00464.x 12930214

[23] Xuan Z , Chaloupka FJ , Blanchette JG , Nguyen TH , Heeren TC , Nelson TF , The relationship between alcohol taxes and binge drinking: evaluating new tax measures incorporating multiple tax and beverage types. Addiction 2015;110(3):441–50. 10.1111/add.12818 25428795 PMC4441276

[24] Chaloupka FJ , Grossman M , Saffer H . The effects of price on alcohol consumption and alcohol-related problems. Alcohol Res Health 2002;26(1):22–34. 12154648 PMC6683806

[25] Rehm J , Taylor B , Mohapatra S , Irving H , Baliunas D , Patra J , Alcohol as a risk factor for liver cirrhosis: a systematic review and meta-analysis. Drug Alcohol Rev 2010;29(4):437–45. 10.1111/j.1465-3362.2009.00153.x 20636661

[26] Herttua K , Mäkelä P , Martikainen P . Changes in alcohol-related mortality and its socioeconomic differences after a large reduction in alcohol prices: a natural experiment based on register data. Am J Epidemiol 2008;168(10):1110–8, discussion 1126–31. 10.1093/aje/kwn216 18718894 PMC2727242

[27] May PA . Alcohol policy considerations for Indian reservations and bordertown communities. Am Indian Alsk Native Ment Health Res 1992;4(3):5–59, discussion 71–5, 95–100. 10.5820/aian.0403.1990.5 1504172

[28] Landen MG . Alcohol-related mortality and tribal alcohol legislation. J Rural Health 1997;13(1):38–44. 10.1111/j.1748-0361.1997.tb00832.x 10167764

[29] Corrao G , Ferrari P , Zambon A , Torchio P . Are the recent trends in liver cirrhosis mortality affected by the changes in alcohol consumption? Analysis of latency period in European countries. J Stud Alcohol 1997;58(5):486–94. 10.15288/jsa.1997.58.486 9273913

[30] Kanny D , Brewer RD , Mesnick JB , Paulozzi LJ , Naimi TS , Lu H . Vital signs: alcohol poisoning deaths - United States, 2010-2012. MMWR Morb Mortal Wkly Rep 2015;63(53):1238–42. 25577989 PMC4646044

